# Supplementary figures and images for: Programmatic mapping and size estimation of key populations to inform HIV programming in Tanzania
Source: PLoS One. 2020 Jan 30;15(1):e0228618. doi: 10.1371/journal.pone.0228618 (PMC6992209; doi:10.1371/journal.pone.0228618)

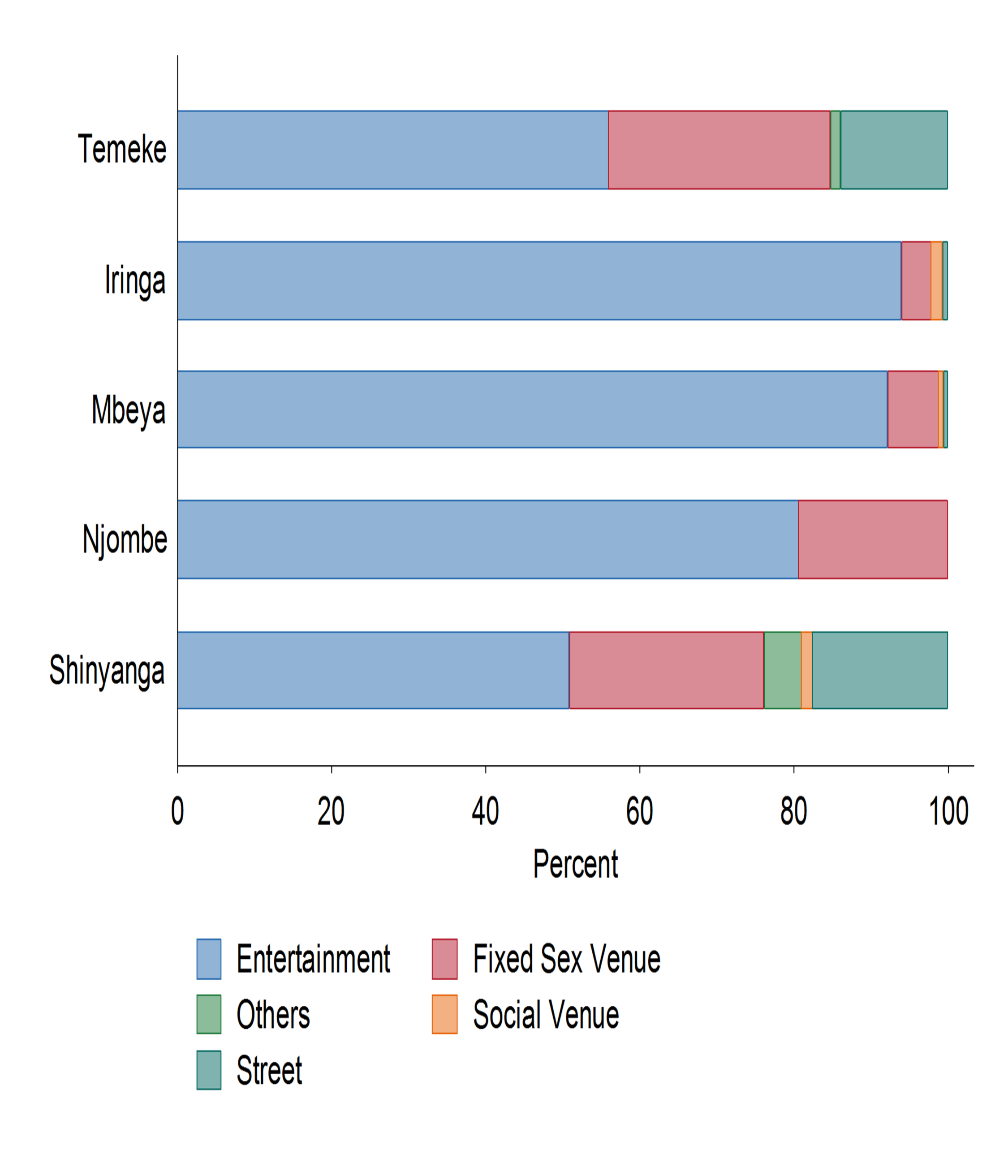

Supplement: S1 Fig — (TIFF) [file pone.0228618.s001.tiff]

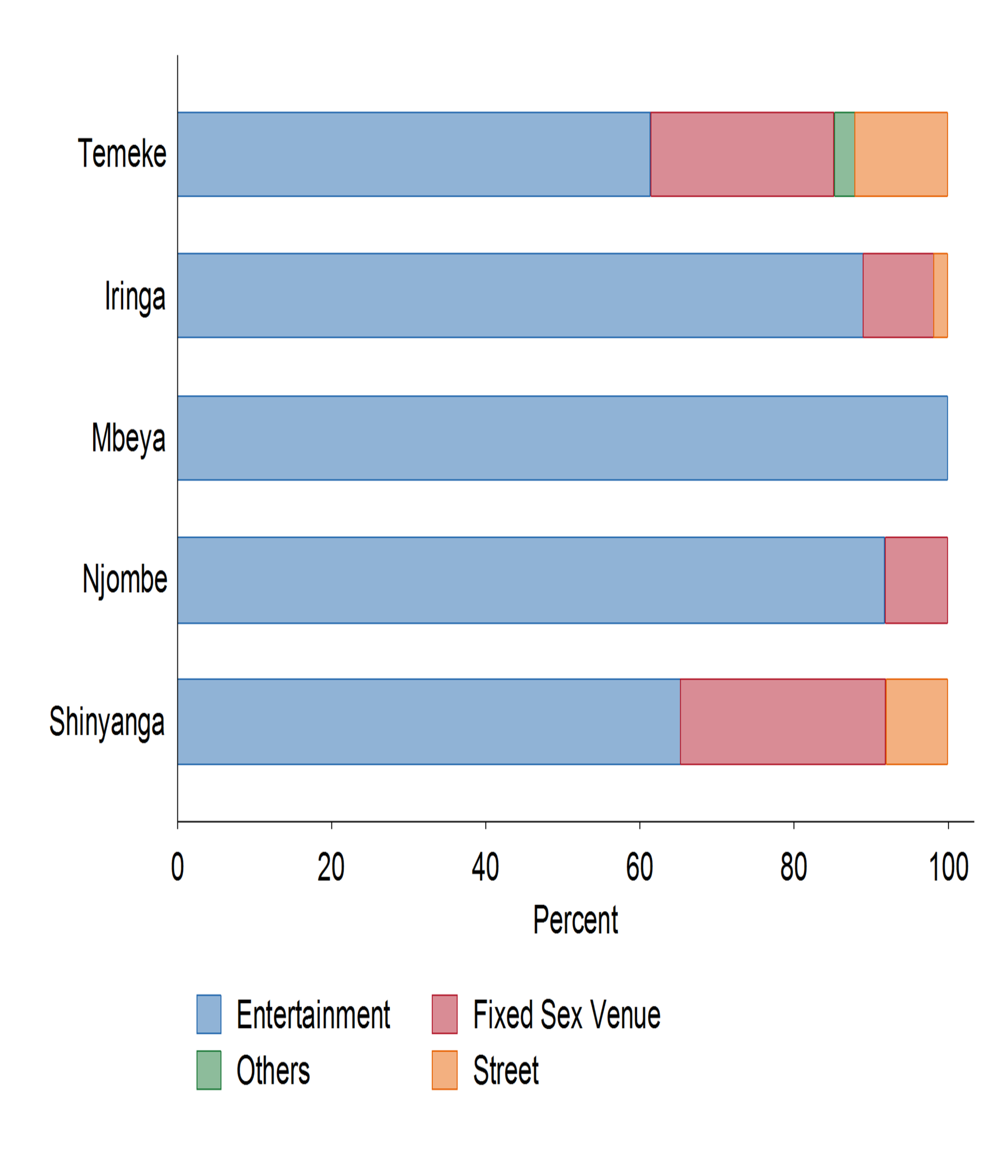

Supplement: S2 Fig — (TIFF) [file pone.0228618.s002.tiff]
