# Supplementary material for: Programmatic mapping and size estimation of key populations to inform HIV programming in Tanzania
Source: PLoS One. 2020 Jan 30;15(1):e0228618. doi: 10.1371/journal.pone.0228618 (PMC6992209; doi:10.1371/journal.pone.0228618)
Supplement: Attachment — Submitted filename: Mapping comments Revised 15jan2020.pdf [file pone.0228618.s003.pdf]

PONE-D-19-24277R1

Programmatic mapping and size estimation of key populations to inform HIV programming in Tanzania, Dr Mwita Wambura

Dear Dr Wambura,

Thank you for submitting your manuscript entitled "Programmatic mapping and size estimation of key populations to inform HIV programming in Tanzania" to PLOS ONE. Your manuscript files have been checked in-house but before we can proceed we need you to address the following issues:

1. Thank you for your response. During our internal evaluation, we noted that the following sentence was removed from your ethics statement

'To ensure that the local administration in areas where the research was done is informed about the study, approvals to work in the study communities was sought from the regional and district health authorities and from the respective ward and village executive officers.'

To ensure that a full description of the ethical considerations taken in this study is documented in your manuscript, we ask that you please provide specific details about the approvals sought from the regional and district health authorities in your study.

Response: "The geographical mapping study received ethical approval from the Medical Research Coordinating Committee (MRCC) of the National Institute for Medical Research (NIMR) in Tanzania (IRB00002514; FWA00002632) with a letter with reference number NIMR/HQ/R.8a/Vol. IX/2086 (attached), and Institutional Review Board of the Johns Hopkins University (IRB 00006668, FWA00000287) with letter dated 15th October 2015 (attached).

The monitoring of research studies is done by MRCC in collaboration with district and regional medical authorities. Therefore, the Sauti project wrote letters to seek permission from the regional and district health authorities. I have attached letters from regional, district and community authorities providing permission. Because of a large number of letters received, we have only attached a sample for your review.

The above statement was deleted because regional and district health authorities are not ethics committees, and therefore, do not provide ethical approvals.

THE UNITED REPUBLIC OF
TANZANIA

National Institute for Medical Research
3 Barack Obama Drive
P.O. Box 9653
11101 Dar es Salaam
Tel: 255 22 2121400
Fax: 255 22 2121360
E-mail: headquarters@nimr.or.tz
NIMR/HQ/R.8a/Vol. IX/2086

Ministry of Health and Social Welfare
6 Samora Machel Avenue
P.O. Box 9083
11478 Dar es Salaam
Tel: 255 22 2120262-7
Fax: 255 22 2110986

16th December 2015

Mr Hally Mahler
JHPIEGO/ Tanzania
P O BOX 9170
DAR ES SALAAM

CLEARANCE CERTIFICATE FOR CONDUCTING MEDICAL RESEARCH IN TANZANIA

This is to certify that the research entitled: Programmatic/ Geographical Mapping of Key Populations in Tanzania, (Mahler H *et al*), whose Local Investigator is Ms Neema Makyao, National Key Populations Coordinator, NACP, MoH &SW, Dar es Salaam, has been granted ethical clearance to be conducted in Tanzania.

The Principal Investigator of the study must ensure that the following conditions are fulfilled:

1. Progress report is submitted to the Ministry of Health and the National Institute for Medical Research, Regional and District Medical Officers after every six months.
2. Permission to publish the results is obtained from National Institute for Medical Research.
3. Copies of final publications are made available to the Ministry of Health & Social Welfare and the National Institute for Medical Research.
4. Any researcher, who contravenes or fails to comply with these conditions, shall be guilty of an offence and shall be liable on conviction to a fine. NIMR Act No. 23 of 1979, PART III Section 10(2).
5. Sites: Dar es Salaam, Dodoma, Morogoro,Arusha, Tanga, Geita, Mwanza, Shinyanga, Iringa, Mbeya Njombe, and Ruvuma Regions.

Approval is for one year: 16th December 2015 to 15th December 2016.

Name: Dr Mwelecele Malecela

Signature
CHAIRPERSON
MEDICAL RESEARCH
COORDINATING COMMITTEE

Name: Prof. Muhammad Bakari Kambi

Signature
CHIEF MEDICAL OFFICER
MINISTRY OF HEALTH, SOCIAL
WELFARE

CC: **RMO**
DED
DMO

JOHNS HOPKINS
BLOOMBERG SCHOOL
of PUBLIC HEALTH

Institutional Review Board Office

615 N. Wolfe Street / Room E1100
Baltimore, Maryland 21205-2179
Phone: 410-955-3193
Toll Free: 1-888-262-3242
Fax: 410-502-0584
Email: jhsph.irboffice@jhu.edu
Website: www.jhsph.edu/irb

FWA #00000287

NOT HUMAN SUBJECTS RESEARCH

DETERMINATION NOTICE

Date: October 15, 2015

To: Hally Mahler, MHS
Jhpiego: JH Prog for Int'l Educ. in Gyn/Ob

Re: **Study Title**: Programmatic and Geographical Mapping of Key Populations in Tanzania**"**
IRB No: 00006668

The JHSPH IRB-FC reviewed the above-referenced new application on **October 7, 2015**. We have determined that the proposed activity described in your application will involve mapping activities to identify the locations/sites where key population members congregate and/or meet casual or paying sexual partners and key informant interviews concerning the places and areas where sex workers and men who have sex with men are seen and congregate. No personal or private information about informants will be collected. Thus, the proposed activity does not qualify as human subjects research as defined by DHHS regulations 45 CFR 46.102, and does not require IRB oversight.

We anticipate that you will follow ethical practices in your interactions with individuals in the community during the course of your project. You are responsible for notifying the JHSPH IRB of any future changes that might involve human subjects and require IRB oversight.

If you have any questions regarding this action, please contact the JHSPH IRB Office at (410) 955-3193 or via email at jhsph.irboffice@jhu.edu.

JP/teb

JAMHURI YA MUUNGANO WA TANZANIA
Ofisi ya Rais
TAWALA ZA MIKOA NA SERIKALI ZA MITAA

MKOA WA DAR ES SALAAM
Anwani ya Simu:
Simu: 2203156/2203158
Unapojibu Tafadhali taja:
Kumb.Na: FA.282/293/01Q/327

Ofisi ya Mkuu wa Mkoa,
3 Barabara ya Rashidi Kawawa,
S.L.P. 5429,
12880, **DAR ES SALAAM**
25 Aprili, 2016

Wakurugenzi,
Halmashauri ya Manispaa,
Ilala, Temeke na Kinondoni,
DAR ES SALAAM.

YAH: KIBALI CHA KUFANYA UTAFITI WA MAKUNDI MAALUM YALIYO KATIKA HATARI KUBWA YA MAAMBUKIZI YA VIRUSI VYA UKIMWI TANZANIA

Tafadhali rejeeni kichwa cha habari hapo juu.

Taasisi ya utafiti wa madawa ya binadamu (NIMR), kituo cha Mwanza wakishirikiana na Kitengo cha kudhibiti maambukizi ya virusi vya **UKIMWI** (NACP), mradi wa SAUTI na Chuo Kikuu cha Sayansi za Tiba Muhimbili (MUHAS) wameomba kufanya utafiti katika Manispaa ya Temeke, Ilala na Kinondoni.

Madhumuni ya utafiti huu ni kupata taarifa ya kuishauri Kitengo cha kudhibiti maambukizi ya virusi vya UKIMWI kuhusu ukubwa wa tatizo na mahali ambapo makundi maalumu yanapatikana ili kuweza kutekeleza miradi ya kuzuia virusi vya UKIMWI kwa jamii.

Nawaleta kwenu ili muweze kuwapa ushirikiano waweze kufanikisha utafiti huu.

Nashukuru.

Dkt.Grace Elias Magembe
k.n.y: **KATIBU TAWALA MKOA**
DAR ES SALAAM

Nakala: Waganga Wakuu,
Halmashauri ya Manispaa,
Ilala, Temeke na Kinondoni,
DAR ES SALAAM.

JAMHURI YA MUUNGANO WA TANZANIA
OFISI YA RAIS
TAWALA ZA MIKOA NA SERIKALI ZA MITAA

MKOA WA NJOMBE
Simu Na: (026) 2782912
2782913
Nukushi: (026) 2782914
Barua pepe: rc@njombe.go.tz
ras@njombe.go.tz
info@njombe.go.tz
Unapojibu tafadhali
Kumb. taja:AB.24/257/07/34

Ofisi ya Mkuu wa Mkoa
S.L.P. 668
NJOMBE.
26/8/2016

Mkurugenzi Mtendaji,
Halmashauri ya Wilaya Ludewa na Wanging'ombe, Njombe (W)

Mkurugenzi,
Halmashauri ya Mji Njombe

YAH: KUTAMBULISHA KWAKO WATAFITI TOKA NIMR WAKISHIRIKIANA NA SHIRIKA LA JHPIEGO SAUTI

Rejea somo hapo juu,

Ofisi ya Mkuu wa Mkoa wa Njombe imepokea barua yenye Kumb.Na.189/Jhp-Sauti/FY16 ya tarehe 15/8/2016 kutoka shirika la Jhpiego SAUTI inayohusu utafiti uwepo wa makundi maalumu yaliyo katika hatari zaidi ya kupata maambukizi ya VVU (Key population) na maeneo wanakopatikana ndani ya Mkoa wa Njombe (mapping).Utafiti huu utafanyika kwa kushirikiana na NIMR kuanzia tarehe 29/8/2016 hadi tarehe 30/11/2016 katika Halmashauri za Wanging'ombe,Ludewa na Njombe Mji.

Kwa barua hii ninaomba uwaruhusu watafiti hawa kufanya kazi hii katika maeneo tajwa hapo juu.Ninaambatanisha nakala ya ruhusa ya kufanya utafiti huu (certificate of clearance) toka NIMR na ratiba ya utafiti huu.

Nawatakia kazi njema,

Ekessy.
Dkt.Eusebi Kessy
KNY: KATIBU TAWALA (M)
NJOMBE

Nakala: Katibu Tawala (M)-aione katika jalada

Bu: SAUTI Project

THE UNITED REPUBLIC OF TANZANIA

PRESIDENT'S OFFICE

REGIONAL ADMINISTRATION AND LOCAL GOVERNMENT

MBEYA REGION
TELEGRAM: "REGCOM"
Telephone No: 025-2504045
Fax No.025-2504243
Email: *rasmbeya@pmoralg.go.tz*

REGIONAL COMMISSIONER'S OFFICE
P.O. Box 754,
MBEYA.

In reply please quote:

Ref. No. DC.10/109/01/44

25th April, 2016

District Administrative Secretary,
Mbeya, Rungwe, Mbarali, Kyela nad Chunya,
MBEYA REGION.

National Institute for Medical Research – Mwanza
Received... [illegible]
Date... 5/5/16

Ref: **PERMISION TO GIVE PERMITS**

The heading captioned above refers.

The National Institute for Medical research (NIMR) Mwanza Centre in collaboration with the National AIDS Control Program (NACP) SAUTI Program and Muhimbili University of Health and Allied Science (NUHAS) are conducting a study, titled "Mapping and Enumeration of Key Population in Tanzania. The study being carried in Mbeya Region, the Regional Administrative Secretary have authorized you to avail the aforemention Institute permits to conduct their study in your District on behalf of Regional Administrative Secretary.

Do please accord them.

I. E .Nganya
For: **REGIONAL ADMINISTRATIVE SECRETARY,**
MBEYA.

CC. National Institute for Medical Research, - (Please be Informed)
Mwanza Reseach Centre,
P.O. Box 1462,
MWANZA.

Scanned by CamScanner

THE UNITED REPUBLIC OF TANZANIA
PRESIDENT'S OFFICE
REGIONAL ADMINISTRATION AND LOCAL GOVERNMENT

Telegrams "REGCOM" Shinyanga
Tel. No. 028 –2762222
Fax No: 028 – 2762310
E-Mail: ras.shinyanga@pmoralg.go.tz.

Regional Commissioner's Office,
P O. Box 320,
Shinyanga:

Ref. No. EC.81/271/01/32

10th May, 2016

District Exectuve Director
Kishapu, Msalala, Ushetu and Shinyanga

Municipal Director
Shinyanga

Town Director
Kahama

Re: MAPPING AND ENUMERATION OF KEY POPULATION IN TANZANIA

Reference is made for the subject captioned above.

The National Institute for Medical Research (NIMR), Mwanza Centre in collaboration with the National AIDS Control Program (NACP) SAUTI program and Muhimbili University of Health and Allied Sciences (MUHAS) are conducting a study titled "Mapping and Enumeration of Key Population in Tanzania" in a number of selected regions in Tanzania including Shinyanga region. The aim of the study is to inform the National AIDS Control program on the size of key populations in the country, their HIV risk profiles and locations where they can be found so as to effectively implement HIV/AIDS interventions in this population. The study has been granted an approval by NIMR ethical review board. A copy of the ethics approval is herewith attached for your attention.

With this letter I am introducing to you a group of scientist for the said undertaking.

It is my hope that you will offer your maximum support for this activity.

Yours sincerely,

Dr. Ntuli A. Kapologwe
For: REGIONAL ADMINISTRATIVE SECRETARY
SHINYANGA

OFISI YA RAIS

TAWALA ZA MIKOA NA SERIKALI ZA MITAA
HALMASHAURI YA WILAYA YA IRINGA

Barua zote ziandikwe kwa:
Mkurugenzi Mtendaji wa Wilaya
Simu nambari :2702828/2702585
Fax Na. 2701776/2700580/2701755
e-mail: drignas.mlowe@iringadc.go.tz

IDARA YA AFYA NA USTAWI WA JAMII (W),
S.L.P. 108,
IRINGA.
Tanzania

Kumb.Na. DMO/IR/T.40/2/115

22 SEPTEMBA, 2016

Afisa Mtendaji wa Kata,
Kata ya Maboba, Wasa na Ilolompya
H/Wilaya ya Iringa.

YAH: KIBALI CHA UTAFITI WA MAKUNDI MAALUM

Husika na somo hilo hapo juu.

Halmashauri imepokea barua kutoka kwa Katibu Tawala (M) yenye somo hilo hapo juu; kibali hicho kinaanza kuanzia tarehe 16/12/2015 mpaka tarehe 15/12/2016.

Kwa barua hii naomba uwapokee watatifi kutoka NIMR kwa ajili ya utafiti wa makundi ya watu maalum.

Naomba wapewe ushirikiano,

Samwel O. Nkya
KAIMU MGANGA MKUU (W),
HALMASHAURI YA WILAYA YA IRINGA.

Nakala kwa: Wah. Madiwani,
Kata za Maboba, Wasa na Ilolompya,
H/Wilaya ya Iringa. – kwa taarifa.

DISTRICT MEDICAL OFFICER
IRINGA DISTRICT COUNCIL
P. O. BOX 108 - IRINGA

THE UNITED REPUBLIC OF TANZANIA
PRISIDENT'S OFFICE
REGIONAL ADMINISTRATION AND LOCAL GOVERNMENT

MBEYA REGION
TELEGRAM: "ADMIN".
Telephone No: 2502002
In reply please quote:

DISTRICT COMMISSIONER'S OFFICE,
P.O. Box. 255,
MBEYA.

Ref. No. AB.120/369/01/D/354

28th April, 2016

District Executive Director,
Mbeya District Council,
P.O. Box 599,
MBEYA.

REF: RESEARCH PERMIT

Please refer to the above captioned subject.

The National Institute for Medical research (NIMR) Mwanza Centre in collaboration with the National AIDS Control Program (NACP) SAUTI Program and Muhimbili University of Health and allied Science (MUHAS) are conducting a study.

At the moment they conducting research on "Mapping and Enumeration of Key Population in Tanzania". A case study of Mbeya Rural.

Please assist them accordingly.

Tumsifu Ilomo
For: **DISTRICT ADMINISTRATIVE SECRETARY**
MBEYA

c.c. National Institute for Medical Research,
Mwanza Research Centre,
P.O. Box 1462,
MWANZA. – Please be informed.

JAMUHURI YA MUUNGANO WA TANZANIA
OFISI YA RAIS
TAWALA ZA MIKOA NA SERIKALI ZA MITAA

Anuani ya simu "ADMIN"
Simu ya mdomo 025 2552036
FAX NO 0252552421 .

Ofisi ya Mkuu wa Wilaya Rungwe,
S.L.P 34,
TUKUYU.

12/05/2016

Unapojibu taja:-
Kumb.AB.353/574/01/146

Afisa Mtendaji Kata ya
……………………………………….
WILAYA YA RUNGWE.

Yah: IDADI NA MAHALI YAPATIKANAPO MAKUNDI MAALUM NCHINI TANZANIA

Tafadhali rejea mada tajwa hapo juu.

Nakitambulisha kwako kituo cha Utafiti (NIMR) Tawi la Mwanza kwa kushirikiana na Taasisi ya Taifa ya Kuzuia UKIMWI (AIDS) Progrm ya SAUTI na Chuo cha Muhimbili (NUHAS) Inafanya utafiti unaohusu **IDADI NA MAHALI YAPATIKANAPO MAKUNDI MAALUM NCHINI TANZANIA**

Kibali kitatumika kuanzia tarehe 15/5/201 mpaka tarehe 15/6/2016.

Tafadhali mpeni ushirikiano.

S. Mawila.
Susan E. Mawila
Kny: KATIBU TAWALA WILAYA
RUNGWE.

Kny ATIBU TAWALA WILAYA
RUNGWE

Nakala: Mkuu wa Wilaya
RUNGWE- Aione ndani ya jalada.

" Katibu Tawala Wilaya
WILAYA YA RNGWE. – Aione ndani ya jalada

" Mkurugenzi Mtendaji,
Halmashauri ya Wilaya
RUNGWE. - Kwa taarifa.

HALMASHAURI YA WILAYA YA USHETU
Barua zote zipelekwe kwa Mkurugenzi Mtendaji (W)

Mkoa wa Shinyanga.
Simu : 028-2710110
Fax 028-2710100
mkurugenzi@ushetucoucil.go.tz

Ofisi ya Mkurugenzi Mtendaji (W),
S. L. P. 50,
USHETU.

Unapojibu Tafadhali taja:
Kumb. M.10/15/182

Tarehe **31/05/2016**

Afisa Mtendaji wa Kata,
Kata ya Igwamanoni
Ushetu.

Imepitishwa
16/06/2016

AFISA MTENDAJI WA KATA
KATA YA IGWAMANONI
USHETU

YAH: KUTAMBULISHA WATAMBUZI WA MAENEO MUHIMU YANAYOPATIKANA MAKUNDI MAALUMU KWA AJILI YA KUPAMBANA NA VVU/UKIMWI

Husikeni na mada tajwa hapo juu.

Taasisi ya utafiti wa magonjwa ya binadamu (NIMR) kanda ya Mwanza kwa kushirikiana na Mpango wa Taifa wa Kudhibiti UKIMWI (NACP) kupitia mradi wa SAUTI litaendesha zoezi la utambuzi wa maeneo muhimu yanayopatikana makundi maalumu (Key Population). Lengo la utambuzi huu ni kupata taarifa muhimu zitakazosaidia katika kupambana na VVU/UKIMWI kwa maeneo yaliyo katika hatari zaidi.

Kwa barua hii, mnaombwa kutoa ushirikiano mkubwa kwa watambuzi watakaotumwa na taasisi ya NIMR- Mwanza ili malengo ya zoezi hili yafikiwe kwa maendeleo ya Halmashauri yetu, Wilaya, Mkoa na Taifa kwa ujumla.

Tarehe rasmi ya zoezi mtajulishwa na mdau wetu.

Nawatakia utekelezaji mwema.

Chilumba
....
Isabela O. Chilumba
Mkurugenzi Mtendaji
Halmashauri ya Wilaya
Ushetu

MKURUGENZI MTENDAJI (W)
USHETU

Nakala
Mheshimiwa diwani,
Kata ya Igwamanoni,
Ushetu.

TEMEKE MUNICIPAL COUNCIL

[All letters should be addressed to the Municipal Director]

Tell: +255 22-2851054
Fax: +255 22-2850640
E- mail: temekemanispaa@tmc.go.tz
website: www.tmc.go.tz

P.O.Box: 46343,
Mandela Road
DAR ES SALAAM,
TANZANIA.

Ref. No. TMC/MD/ U.21/30

Date: 27/04/2016

WEOs
TEMEKE MUNICIPALITY

RE: RESEARCH PERMIT DR. AMANI SHAO & OTHERS

Please refer to the heading above

This is to inform you that, permission is granted to the above mentioned ~~student~~/Researcher from N.I.M.R to conduct research on Makundi Maalum yaliyo ktk Hatari Maambukizi wa case study of TMC.
This permit will effect from the date of this letter.

Please give with necessary assistance.

Tafadhali wapokee na uwape ushirikiano
18/5/2016

AFISA MTENDAJI WA KATA
TANDIKA

Julie Moe
For: **MUNICIPAL DIRECTOR**
TEMEKE

For: MUNICIPAL DIRECTOR
TEMEKE

Copy to: - Student/Researcher DR AMANI SHAO & OTHERS
From NIMR

HALMASHAURI YA MANISPAA YA SHINYANGA

Simu. **028-2763213**
028-2762534
Fax: 028-2763750
E-mail: municipalshy@yahoo.com

Ofisi ya Mkurugenzi wa Manispaa,
S.L.P. 28,
SHINYANGA

Tarehe: 18 .07.2016

Unapojibu Tafadhali Taja

Kumb. Na. SHY/MC/M.10/20/04

Afisa Mtendaji
Kata ya ...Ngokolo...
S.L.P 28
SHINYANGA.

Imeritishwa Hussein 18/7/2016

Ushirikiano utakuwepo

MTENDAJI WA KATA
KATA YA NGOKOLO
MANISPAA-SHINYANGA

YAH: KUTAMBULISHA WATAMBUZI WA MAENEO MUHIMU YANAYOPATIKANA MAKUNDI MAALUM KWA AJILI YA KUPAMBANA NA VVU/UKIMWI

Husika na somo tajwa hapo juu.

Taasisi ya utafiti wa magonjwa ya binadamu (NIMR) kanda ya Mwanza kwa kushirikiana na Mpango wa Taifa wa kudhibiti UKIMWI (NACP) kupitia mradi wa SAUTI litaendesha zoezi la utambuzi wa maeneo muhimu yanayopatikana makundi maalum (Key Population).Lengo la utambuzi huu ni kupata taarifa muhimu zitakazosaidia katika kupambana na VVU/UKIMWI kwa maeneo yaliyo katika hatari zaidi.

Kwa barua hii,mnaombwa kutoa ushirikiano mkubwa kwa watambuzi watakaotumwa na taasisi ya NIMR- Mwanza ili malengo ya zoezi hili yafikiwe kwa maendeleo ya Halmashauri yetu,Wilaya,Mkoa na Taifa kwa ujumla.

Nawatakia utekelezaji mwema.

Lewis Kalinjuna
Mkurugenzi wa Manispaa
Manispaa ya Shinyanga

MKURUGENZI WA MANISPAA
HALMASHAURI YA MANISPAA
SHINYANGA

HALMASHAURI YA WILAYA YA MUFINDI

(Barua zote zitumwe kwa Mkurugenzi Mtendaji Wilaya)

S.L.P 223,
SIMU: 026-2772614
FAX: 026-2772070
Email: dedmufindi@gmail.com

Kumb.Na. HW/MUF/ S.50/17/25 **14 Septemba, 2016**

Watendaji wa Kata za,
Nyololo, Luhunga, Igombavanu, Kasanga,
Ifwagi na Makungu
Halmashauri ya Wilaya ya Mufindi

YAH: KUITAMBULISHA TIMU YA WAFANYA UTAFITI NA TATHMINI KUHUSU MAKUNDI MAALUM KATIKA KATA YAKO

Kichwa cha Habari hapo juu chahusika.

Timu ya wafanyakazi kutoka NIMR wakishirikiana na Sauti Project, Chuo Kikuu Mannitoba na MUHAS wanafanya utafiti na tathmini ya mambo mbalimbali yanayohusu Makundi Maalumu katika Mikoa 12 ya Tanzania Bara ikiwemo Mkoa wa Iringa.

Katika Mkoa wa Iringa, Tafiti hii pia itafanyika katika Halmashauri ya Wilaya ya Mufindi na katika Kata yako. Hivyo basi kwa barua hii unatakiwa uwapokee watafiti hawa na kuwapatia ushirikiano katika kata yako.

Dr.Fredrick Mugarula
Kny Mkurugenzi Mtendaji Wilaya
MUFINDI

MGANGA MKUU WA WILAYA
MUFINDI

OFISI YA KATA
S L P 129
MAFINGA
26-09-2016

MWENYEKITI WA KIJIJI
AFISA MTENDAJI

YAH: KUWATAMBULISHA WATU WA UTAFITI
WA NIMR

Husika na somo hilo. Watajwa hapo juu wape
Ushirikiano wa kina ili kufanikisha lengo la utafiti

Natanguliza shukrani

AFISA ELIMU KATA
KATA YA IGOMBAVANU

for WEO
IGOMBAVANU

0754557567
E.B. SAMBAYEMA

AFISA MTENDAJI WA KATA,
KATA YA KALOBE,
S.L.P 149,
MBEYA.

MAAFSA WATENDAJI WA MITAA,
KATA YA KALOBE;
P.O BOX 149,
MBEYA.

YAH: BARUA YA UTAMBULISHO.

Husika na kichwa cha barua hapo juu. Napenda kuwatambulisha wanafunzi kutoka shirika la utafiti (MIMR) kituo cha Mwanza. Pamoja na utambulisho huo watafanya utafiti kuhusu makundi maalum yaliyopo kwenye athari ya maambukizi ya virusi vya ukimwi

Naomba muwape ushirikiano.

Afisa mtendaji wa kata ya kalobe

Mwanza.

AFISA MTENDAJI
KATA YA KALOBE
HALMASHAURI YA JIJI
S.L.P. 149 MBEYA
